# Effects of the Dark Septate Endophyte (DSE) *Exophiala pisciphila* on the Growth of Root Cell Wall Polysaccharides and the Cadmium Content of *Zea mays* L. under Cadmium Stress

**DOI:** 10.3390/jof7121035

**Published:** 2021-12-02

**Authors:** Yao Xiao, Meng-Xue Dai, Guang-Qun Zhang, Zhi-Xin Yang, Yong-Mei He, Fang-Dong Zhan

**Affiliations:** College of Resources and Environment, Yunnan Agricultural University, Kunming 650201, China; xiao.yao@mail.huji.ac.il (Y.X.); dmx1443755433@163.com (M.-X.D.); zhang12241994@163.com (G.-Q.Z.); zhixinyang2021@126.com (Z.-X.Y.); heyongmei06@126.com (Y.-M.H.)

**Keywords:** dark septate endophytes, cadmium stress, root cell wall, polysaccharide component

## Abstract

This paper aims to investigate the mechanism by which dark septate endophytes (DSEs) enhance cadmium (Cd) tolerance in there host plants. Maize (*Zea mays* L.) was inoculated with a DSE, *Exophiala pisciphila*, under Cd stress at different concentrations (0, 5, 10, and 20 mg·kg^−1^). The results show that, under 20 mg/kg Cd stress, DSE significantly increased maize biomass and plant height, indicating that DSE colonization can be utilized to increase the Cd tolerance of host plants. More Cd was retained in DSE-inoculated roots, especially that fixed in the root cell wall (RCW). The capability of DSE to induce a higher Cd holding capacity in the RCW is caused by modulation of the total sugar and uronic acid of DSE-colonized RCW, mainly the pectin and hemicellulose fractions. The fourier-transform spectroscopy analysis results show that carboxyl, hydroxyl, and acidic groups are involved in Cd retention in the DSE-inoculated RCW. The promotion of the growth of maize and improvement in its tolerance to Cd due to DSEs are related to restriction of the translocation of Cd from roots to shoots; resistance of Cd uptake Cd inside cells; and the increase in RCW-integrated Cd through modulating RCW polysaccharide components.

## 1. Introduction

Cadmium (Cd) is a widespread environmental pollutant with acute and chronic toxicity in plants and animals [1]. Cd is taken up by plants, and can bind directly with protein functional groups, leading to changes in their conformation and functioning [2], resulting in phytotoxicity, such as growth reduction [3,4]; damage to the photosynthetic apparatus or modulation of chlorophyll fluorescence [5,6,7,8,9,10]; and changes to plant physiological and biochemical mechanisms [11,12,13]. Cd can cause the overaccumulation of reactive oxygen species (ROS), leading to oxidative stress and resulting in membrane damage, electrolyte leakage, an increased mutation rate, and reduced efficiency of various metabolic processes [14]. Antagonistic interactions between Cd and mineral nutrients lead to nutrient deficiencies [15]. Additionally, Cd accumulates in the edible parts of plants through root absorption and translocation, which endangers human health through the contamination of the food chain, causing broad concern [16].

Root cell walls (RCWs) serve as the first barrier against Cd stress. Recent evidence indicates that many types of plants alleviate heavy metal stress by binding heavy metals to the cell wall [17,18]. For example, large amounts of Cd in many crops, such as maize [19], soybean [20], and rice [21], and wheat [22], were found to be fixed in the RCW, restricting them from the protoplast. Cd retention in the cell wall is considered to be an effective detoxification mechanism due to the reduced Cd accumulation in the shoot [23]. Cell wall thickening is a common phenomenon that has been observed in many studies. For instance, Cd in soybean roots was fixed in thickened RCW, enhancing fixation through increased cellulose and lignin [20]. Under aluminum (Al) stress, the lignin synthesis pathway of rice roots is enhanced, leading to cell wall thickenings to immobilize the Al [24]. In addition to blocking heavy metal ions via RCW thickening, many researches claimed that more Cd-integrated RCW is involved in binding heavy metal ions in cell wall polysaccharides [25,26,27]. RCWs are composed of three components: the intercellular layer, the primary wall, and the secondary wall. The wall is a complex mainly composed of polysaccharides, such as pectin (PE), hemicellulose (HE), and cellulose, and their chemical characteristics are related to functional groups, such as carboxyl (-COOH), hydroxyl (-OH), and sulfhydryl groups (-SH) [28,29]. It is widely accepted that pectin methylesterase can catalyze pectin demethylation, reduce the methylation degree of pectin, increase the number of free carboxyl groups, and improve the binding ability of metal ions [26,30]. PE and HE are already accepted to play essential roles in binding divalent or trivalent heavy metal cations which principally rely on the number of acidic mucopolysaccharides and uronic acids. These cell wall components provide binding and adsorption sites for heavy metals before crossing the cell membrane leading to less plant heavy metal accumulation in the cytoplasm [18,29,31,32,33,34,35,36,37,38]. Previous experiments indicate that the content of total sugars and uronic acid in the PE and HE of the cell wall increase significantly under Al and Cu stress [24,39], and an experiment found that high lead ion stress impacts the homogalacturonan content of the uronic acid in *Helianthus annuus* L. root zones [40]. Xylogalacturonan from pectic uronic acid was proven to exert a vital role in increasing the Cd tolerance of *Medicago sativa* [38]. Chen et al. (2013) [41] found that the adsorption of Cd is closely related to the RCW of woody phytoremediation species, *Salix jiangsuensis* J172, suggesting that HE significantly reduced Cd accumulation, while PE and cellulose positively impacted Cd adsorption and cellulose played a major role. Currently, the relationship between Cd accumulation and cell wall components in maize requires further investigation.

Dark septate endophytes (DSEs) generally refer to small fungi that colonize plant roots. The hyphae are dark and have obvious transverse septa, which widely colonize the epidermis, cortex, and intercellular spaces of healthy plant root, and do not cause evident plant disease [42]. DSEs widely exist in stressful environments, such as areas of drought, cold, high-altitude, and heavy metal contamination, and enhance the tolerance of host plants to abiotic stress and show important ecological functions [43,44,45]. DSEs extend mucilaginous hyphae to promote the transportation of water and nutrients in arid environments, helping rice and sorghum survive in arid environments and improving their drought resistance [46,47]. The DSE–host interaction is crucial to plant survival [48]. In particular, the DSE strain, *Exophiala pisciphila*, which was isolated from heavy metal contaminated soil, has a strong tolerance to heavy metals under in vitro culture conditions [49]. In Cd-contaminated areas, *E. psciphila* widely colonizes in the roots of wild plants and the DSEs can enhance the host’s tolerance to this adverse environment. The effects of *E. psciphila* inoculation on plant development are salutary under heavy metal stress. They can form a mutualistic symbiotic relationship with plants [50]. The physiological response, and biological roles of DSEs in alleviating Cd toxicity have been investigated in many studies. For example, He [51] found that the ability of DSEs to increase maize Cd tolerance is related to the regulation on phytohormone balance and photosynthetic activities in maize leaves. Wang [52] found that, under Cd stress, DSE inoculation significantly enhanced the activities of antioxidant enzymes and low-molecular-weight antioxidants. Similar outcomes were found in other studies [53,54]. However, the mechanisms involved in enhancing the Cd tolerance of the so-called “first barrier”, the cell wall, have not been elucidated. Moreover, DSEs influences the heavy metal content of host plants differently, depending on the type and concentration of heavy metals in the substrate. For example, the DSE strain Falciphora oryzae conferred and improved Cd tolerance to rice, decreasing Cd accumulation in roots and translocation to shoots [55]. He [19] found that DSEs alter root traits and restrict Cd migration from the roots to the shoots of maize. As noted above, we know that DSEs can improve the Cd tolerance of host plants and wanted to expand our knowledge on how DSEs affect the Cd distribution of maize under Cd stress and whether the DSE-inoculated cell wall plays a major role as the first barrier to affect the distribution of Cd in host plants. Studies have shown that arbuscular mycorrhizal fungi (AMF) can enhance the sequestration of Cd in the cell wall and consequently promote the holding capacity of Cd in plant roots by affecting the components of RCW polysaccharides [26,56]. Therefore, we speculate that DSE may also increase the fixation of Cd by affecting the polysaccharide composition of the cell wall.

The pot experiment was conducted with *Exophiala pisciphila*, a DSE strain isolated from a lead–zinc mining area, which is highly resistant to Cd stress. A previous study found that DSE colonization in maize roots significantly increased Cd accumulation in the roots under 5 and 20 mg·kg^−1^ treatments [51]. Therefore, under Cd stress (0, 5, 10 and 20 mg·kg^−1^), the effects of DSEs on maize growth, polysaccharide components of the RCW, and Cd content were studied, and the functional groups of polysaccharides of the RCW were studied by Fourier transform infrared spectroscopy (FTIR) to enrich the understanding of the physiological and ecological functions of DSEs. The scientific hypothesis put forward is that DSEs alter the polysaccharide components of the RCW and improve the Cd tolerance of plants.

## 2. Materials and Methods

### 2.1. Soil and Biological Materials

The DSE, *E. pisciphila* H93 (accession number ACCC32496), was isolated from the roots of *Arundinella bengalensis* (Poaceae) growing naturally in an old mine smelting site in Huize County, Yunnan Province, southwest China (103°36′ E, 26°55′ N). The fungus is a dominant colonizer of the roots of wild plants and is preserved in the Agricultural Culture Collection Center of China. It was selected as a DSE inoculant due to its previously examined beneficial functions in symbiosis and extreme tolerance to metal ions [57]. Sterile silica sand culture substrate was used as the culture medium (Zhiyuan Reagent Co., Ltd., Tianjin, China; autoclaved for 2 h at 121 °C three times at two-day intervals).

The test maize is the main locally cultivated variety (Huidan No. 4), which is considered a variety with high Cd tolerance and low Cd accumulation [58]. Full maize seeds of the same size were selected before seed sowing. Huidan No. 4 seeds were surface-sterilized by immersion in 75% ethanol (Damao Chemical Reagent Co., Ltd., Tianjin, China) for 10 min and then in 10% sodium hypochlorite for 10 min. After surface sterilization of the seeds, they were rinsed with sterile water for 4–5 times. Subsequently, the seeds were sown in a sterilized Petri dish (150 mm) and incubated in the dark at 28 °C for three days. Sterile seedlings with consistent growth were selected when the seeds germinated and were approximately 1 cm.

### 2.2. DSE Cultivation and Pot Experiment

When the seeds germinated and formed 1-cm seedlings, the DSE and maize seedlings were inoculated in the symbiotic culture medium. Each medium was planted with two maize seedlings, and then inoculated with two fungal disks (Φ 0.5 cm) cut from a fresh culture of *E. pisciphila* H93 PDA (14daysold) or, for a control treatment, two autoclaved fungal disks (Φ 0.5 cm). The eight treatments (with/without DSE + 4 levels of [Cd^2+^]) were each replicated 16 times, resulting in 128 maize seedlings. The media were placed in a phytotron with a 10 h photoperiod (1000–8000 l×) at 25/15 °C (day/night) and 75% humidity for 14 days.

Thirty-two plastic pots, with 25 cm diameter and 20 cm depth, were surface-sterilized with 75% ethanol before being divided into four groups and supplemented with a series of Cd^2+^ treatments (0, 5, 10, 20 mg kg^−1^ Cd^2+^; CdCl_2_·2.5H_2_O, Damao Chemical Reagent Co., Ltd., Tianjin, China). In each pot, 5 kg of sterile silica sand was poured into each pot, and four maize seedlings inoculated with/without DSE were implanted. Each treatment was repeated four times for a total of 32 pots. The seedlings were cultured in the greenhouse under natural light with a temperature varying between 10–15 °C. Two hundred milliliters of 50% Hoagland nutrient solution was watered into each pot every 5 days. The soil moisture content (15%) was maintained during the experiment. To make each plant of the crop have a certain nutritional area, after 4 days, the redundant seedlings were removed, and only one with better growth was left in each pot. Forty-five days after the transplant, the plant height (form ground to shoot tip) was measured with a graduated rule and the root and shoot tissue were harvested separately for all treatments. The samples were thoroughly washed with deionized water and one root and leaf subsample from each seedling was immediately placed at −80 °C, for subsequent analyses, while the remaining tissue was dried (75 °C, 72 h) to constant weight. The DSE fungal colonization intensity was determined using the gridline intersect method [43]. The cadmium adhered to the root surface was removed by soaking the root for three hours in a solution containing 1% ethylenediaminetetraacetic acid (EDTA, Damao Chemical Reagent Co., Ltd., Tianjin, China) made up in deionized water followed by rinsing five times with deionized water [59]. The shoots were washed 3 times with distilled water. The dried samples were digested with HNO_3_-HClO_4_ (3:1, *v*:*v*, Damao Chemical Reagent Co., Ltd., Tianjin, China) and the Cd concentration was determined using an atomic absorption spectrophotometer (TAS-990, Beijing Puxi Instrument Factory, Beijing, China). Appropriate quality controls included the use of CdCl_2_ as a standard solution.

### 2.3. Cell Wall Extraction

Maize cell wall extraction was conducted, as previously described [60], by crushing cells and extracting intracellular substances. The fresh root samples were homogenized to powder and transferred to 10-mL centrifuge tubes, immersed with 75% ice ethanol and mixed well. After 10 min of standing, the homogenates were centrifuged at 12,000× *g* for 10 min and the supernatant was discarded. This procedure was repeated three times with the addition of three different reagents in order, i.e., ice-clod acetone, methanol–chloroform mixture (*v*/*v* = 1:1), and methanol (Damao Chemical Reagent Co., Ltd., Tianjin, China). Then, the precipitates were freeze-dried. After drying the solid obtained pellets were the crude cell wall which was stored at 4 °C.

### 2.4. Cell-Wall Polysaccharide Extraction

Some modifications were made according to Zhong’s method [61]. The dried crude cell wall sample was weighted, placed in boiling water with ultrapure water buffer (stem cell wall/extract, 5 mg/mL) for 1 h, and centrifuged at 17,000× *g* for 10 min, before pooling the supernatant, which was repeated 3 times. PE was completely extracted, and the supernatant was considered the PE component. The precipitate was subsequently washed twice with deionized water and triple-extracted with 34% sodium hydroxide (NaOH, Damao Chemical Reagent Co., Ltd., Tianjin, China), containing 0.1% sodium borohydride (NaBH_4_, Damao Chemical Reagent Co., Ltd., Tianjin, China) at room temperature, and the supernatant was HE 1. The precipitate was washed twice with deionized water and extracted three times with 24% NaOH (containing 0.1% NaBH_4_. Damao Chemical Reagent Co., Ltd., Tianjin, China) at room temperature, and the supernatant was HE-2.

### 2.5. Characterization of Functional Groups in the RCWs

Fourier transform infrared (FTIR) spectra of the RCW before and after Cd accumulation were acquired using an FTIR spectrometer (Thermo Nicolet Avatar 360, Quebec City, QC, Canada). Two-milligram root samples were ground with 200 mg of potassium bromide (KBr, Damao Chemical Reagent Co., Ltd., Tianjin, China) in an agate mortar until KBr powder adhered to the mortar wall when the particle diameter was approximately 2 μM (diameter 13 mm). The functional groups involved in metal ion biosorption in the RCW were obtained within the range of 400–4000 cm^−1^.

### 2.6. Cd Content Determination

The samples from each cell wall polysaccharide component extraction were wet digested with strong nitric acid. Perchloric acid (HNO_3_:HClO_4_, 4:1, *v*/*v*, Damao Chemical Reagent Co., Ltd., Tianjin, China), at 90 °C for 4 h [62], and an atomic absorption spectrophotometer (TAS-990, Beijing Puxi Instrument Factory, Beijing, China) were used to determine the Cd in the plant and RCW polysaccharide components. The minimum detection limit for Cd was 0.01 mg·kg^−1^ [63]. The standard reference material CdCl_2_ (Damao Chemical Reagent Co., Ltd., Tianjin, China) was used as control.

### 2.7. Determination of the Total Sugar and Uronic Acid Content in the Cell Wall Components

The total sugar in each sample was determined by phenol sulfuric acid colorimetry with glucose (Sigma–Aldrich Reagent Co., LLC., Darmstadt, Germany) as the standard [64]. Briefly, a 200-μL sample from the above cell wall extraction was incubated with 10 μL of 80% phenol and 1 mL of sulfuric acid, which were placed in a boiling water (100 °C) bath for 15 min. Then, the sample was cooled to room temperature, and the absorbance was measured at a wavelength of 490 nm.

The uronic acid of the cell wall was determined by hydroquinone colorimetry [65] using galacturonic acid (Sigma–Aldrich Reagent Co., LLC., Darmstadt, Germany) as the standard. Briefly, 200 μL of the sample from the above cell wall extraction was mixed with 1 mL of sulfuric acid (containing 0.0125 M of Na_2_B_4_O_7_·10H_2_O, Damao Chemical Reagent Co., Ltd., Tianjin, China) at 100 °C for 5 min. After chilling, 20 μL of 3-phenylphenol (0.15%, Sigma–Aldrich Reagent Co., LLC., Darmstadt, Germany) was added to the solution and placed them at room temperature for 20 min. The absorbance was measured spectrophotometrically at 520 nm.

### 2.8. Data Analysis

Microsoft Excel 2013 was used to process the experimental data and calculate the mean and standard deviation. IBM SPSS Statistics v21.0 (Version 23.0, IBM Corp., Chicago, IL, USA) data processing software was used for two-way analysis of variance (ANOVA) and Pearson correlation analysis. Prior to statistical analysis, the normality and homogeneity of variances were confirmed using the Shapiro–Wilkson test and Levene statistics, respectively. The least significant difference (LSD) test was used to test the differences of the mean values at 0.05 and 0.01 levels. Origin Pro 9.0 was used to plot.

## 3. Results

### 3.1. Effects of DSE on Maize Growth under Cd Stress

**Figure 1:**
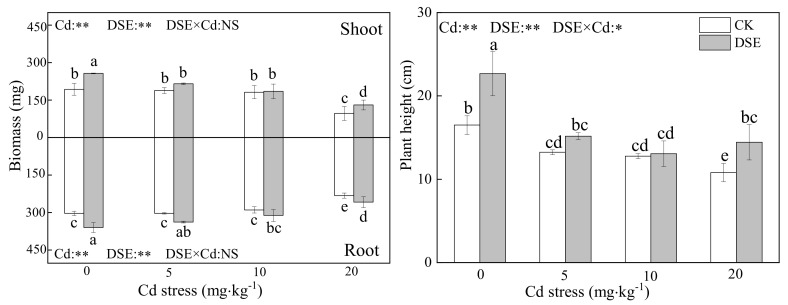
Effects of DSE inoculation on maize biomass and height under Cd stress. Error bar indicates standard deviation. Cd: cadmium treatment; CK: the control of non-inoculation; DSE: *Exophiala* *pisciphila* inoculation; different little letters refer to *p* < 0.05 according to LSD test. “NS”, “*”, and “**” mean no significance, *p* < 0.05 and *p* < 0.01 according to two-way ANOVA, respectively.

All inoculated treatments were successfully colonized by *E. pisciphila* with a range of 15% to 35% colonization and DSE structures were not observed in the roots of non-inoculated maize. As Figure 1 shows, the biomass of the shoots and roots of maize was significantly decreased when the Cd concentration was 20 mg·kg^−1^ for the inoculated and non-inoculated DSE treatments. Compared with the non-inoculated treatment, inoculation with DSE significantly increased maize biomass (roots and shoots) under the four levels of Cd stress, except for the shoot biomass at the 5 and 10 mg·kg^−1^ Cd levels. Moreover, the effect of alleviating plant biomass reduction was more pronounced under the 20 mg·kg^−1^ Cd stress conditions where DSE inoculation increased shoot and root biomass by 23.9 and 23.1%, respectively (Figure 1). Therefore, the incremental toxicity of Cd can be mitigated more in DSE-inoculated maize than in non-inoculated maize.

The height of maize decreased as the concentration of Cd increased in the culture soil, and the non-inoculated plant height decreased by 20%, 23%, and 34.5% at three levels of Cd, respectively. However, in the comparison between non-inoculated and inoculated treatments, the DSE inoculation significantly eased shrinkage of plant height by 36.9%, 14.4%, 2.8%, and 25% in the four levels of Cd experiments, respectively (Figure 1). The two-way ANOVA results showed that there was an interactive effect between DSE inoculation and Cd stress on maize which significantly affected the plant height. The effects of DSE inoculation on plant growth are commonly positive under Cd stress.

### 3.2. Cadmium Content

**Figure 2:**
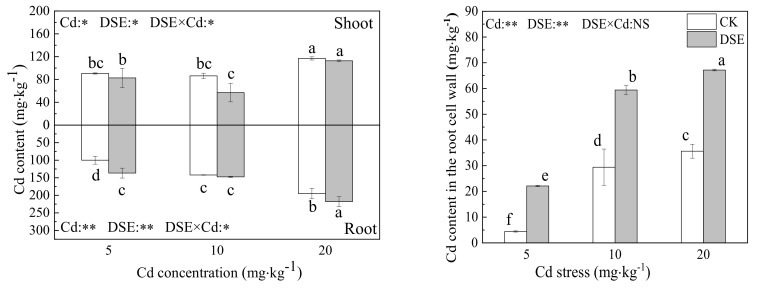
Effects of DSE inoculation on Cd concentration and Cd content of maize shoot, root, and cell wall under Cd stress. Error bar indicates standard deviation. Cd: cadmium treatment; CK: the control of non-inoculation; DSE: *Exophiala pisciphila* inoculation; different little letters refer to *p* < 0.05 according to LSD test. “NS”, “*”, and “**” mean no significance, *p* < 0.05 and *p* < 0.01 according to two-way ANOVA, respectively.

As shown in Figure 2, with elevated Cd in the media, Cd was increasingly absorbed by roots and shoots of both inoculated and non-inoculated treatments, the Cd content in the roots increased significantly, and the Cd content in roots was higher than that in the shoots. Five milligrams per kilogram Cd stress significantly lowered the shoot Cd content, and significantly increased the root Cd content in the inoculated treatments. DSEs may improve the tolerance of maize to Cd by restricting the translocation of Cd ions from the roots to the shoots. DSE inoculation caused an extremely significant increase in the Cd content in the roots by 36.8% under the 5 mg·kg^−1^ Cd treatment and a significant increase of 11.3 % in the 20 mg·kg^−1^ Cd treatment. In contrast,, the shoot Cd content decreased significantly, by 8.7 and 51%, under the 5 and 10 mg·kg^−1^ Cd levels, respectively. DSE inoculation increased the Cd root to shoot ratio from 1.09 to 1.7 under 5 mg·kg^−1^ Cd stress, from 1.65 to 2.57 under 10 mg·kg^−1^ Cd stress, and from 1.67 to 1.93 mg·kg^−1^ Cd stress under 20 mg·kg^−1^ Cd stress. Thus, with DSE inoculation, more Cd remained in the roots while the translocation of Cd from the roots to the shoots was restricted. Similarly, the uptake of Cd in the RCW also increased significantly, according to the increased Cd stress and DSE inoculation which boosted Cd retention in the cell wall.

The two-way ANOVA results showed that there was an interactive effect between the two treatments on the Cd content of shoots and roots. The increased Cd stress stimulated the adsorption of Cd by plants while the Cd content in the roots was increased by DSE inoculation.

### 3.3. Total Sugar Content of Cell-Wall Polysaccharide Components

**Figure 3:**
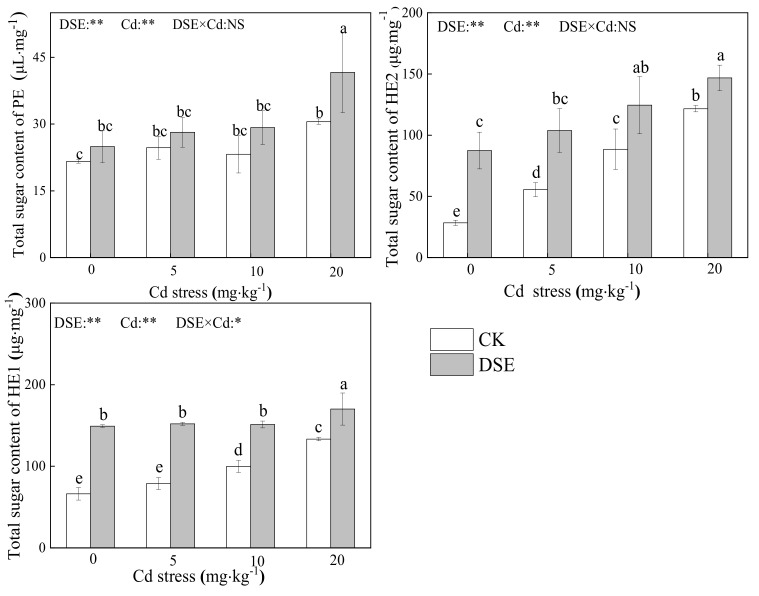
Total sugar content of pectin (PE), hemicellulose–1 (HE1), and hemicellulose–2 (HE2) in maize RCW with DSE-inoculated and Cd stress. Error bar indicates standard deviation. Cd: cadmium treatment; CK: the control of non-inoculation; DSE: *Exophiala pisciphila* inoculation; different little letters refer to *p* < 0.05 according to LSD test. “NS”, “*”, and “**” mean no significance, *p* < 0.05 and *p* < 0.01 according to two-way ANOVA, respectively.

As shown in Figure 3, compared with the blank comparison group, both treatments increased the total sugar content of the cell wall polysaccharide components. Under 20 mg·kg^−1^ Cd stress, the total sugar content of PE, hemicellulose 1 (HE1), and hemicellulose 2 (HE2) in the RCW increased significantly; under no Cd stress, DSE inoculation increased the total sugar of HE1 and HE2 in the RCW significantly. Compared with non-inoculated plants, DSE inoculation significantly increased the total sugar of HE1 and HE2 in the RCW under 5mg·kg^−1^, 10 mg·kg^−1^ and 20 mg·kg^−1^ Cd stress, as well as the PE total sugar at the 20 mg·kg^−1^ Cd level. Overall, in all Cd treatments, DSE inoculation resulted in a significant increase in the total sugar of HE1 and HE2, while a significant increase in PE total sugar was only observed at 20 mg·kg^−1^ Cd stress.

The two-way ANOVA results show that both DSE inoculation and Cd stress treatments had a significant effect on the RCW component carbohydrates content; there was an interaction between DSE inoculation and Cd stress on the total sugar content of HE1. We concluded that DSE could increase the content of RCW polysaccharide components under Cd stress.

### 3.4. The Uronic Acid Content of Cell Wall Components

**Figure 4:**
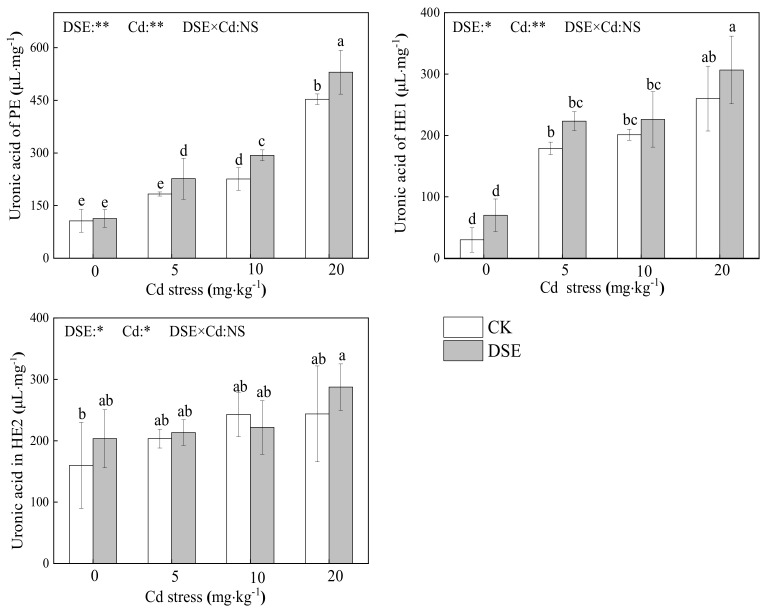
Uronic acid content in pectin (PE), hemicellulose–1 (HE1), and hemicellulose–2 (HE2) of the RCW of inoculated DSE maize with different Cd stress. Error bar indicates standard deviation. Cd: cadmium treatment; CK: the control of non-inoculation; DSE: *Exophiala pisciphila* inoculation; different little letters refer to *p* < 0.05 according to the LSD test. “NS”, “*”, and “**” mean no significance, *p* < 0.05 and *p* < 0.01 according to two-way ANOVA, respectively.

As Figure 4 shows, for each cell wall component, compared to the control group, an increased amount of uronic acid in PE and HE1 was observed under increased Cd exposure levels, and there was little effect on HE2. Furthermore, compared to the non-inoculated controls, inoculation with DSE very significantly improved the uronic acid in PE. Furthermore, the uronic acid increased in HE1 under 5 and 20 mg·kg^−1^ Cd stress and also increased in HE2 under 0 and 20 mg·kg^−1^ Cd stress.

The two-way ANOVA results show that both DSE and Cd stress had a significant effect (*p* < 0.05) on the uronic acid content of cell wall components, respectively. The results showed that inoculation of DSE affected (*p* < 0.05) HE1 uronic acid and HE2 uronic acid, while the uronic acid content of PE was very significantly affected by DSE treatment. Cd supplementation had an extremely significant effect on uronic acid in PE and HE1 and affected (*p* < 0.05) uronic acid in HE2. However, there was no synergistic effect between the two factors (Figure 4).

### 3.5. Characterization of Maize RCW

**Figure 5:**
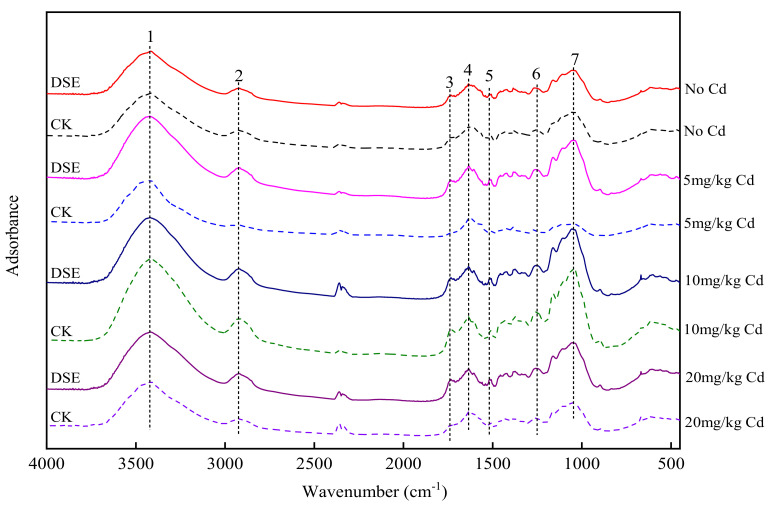
Changes in FTIR spectra of RCWs before and after Cd stress and DSE inoculation. Cd: cadmium treatment; CK: the control of non-inoculation; DSE: *Exophiala pisciphila* inoculation.

As shown in Figure 5, eight FTIR spectra were obtained to decipher which functional groups engaged in Cd uptake in the RCW at three Cd concentration and with DSE inoculation. The broad bands (No. 1) observed at 3520 and 3360 cm^−1^ were assigned to the stretching of the O–H group. The O–H stretching vibrations indicated the presence of free hydroxyl groups and bonded O–H bands of carboxylic acids. The adsorption peaks at 2816–2925 cm^−1^ (No. 2) signify symmetric or asymmetric CH stretching vibrations of aliphatic acids and symmetric stretching vibrations of CH_2_ due to CH bonds of aliphatic acids. The peak observed at 1733 cm^−1^ (No. 3) is the stretching vibration of the C=O bond due to nonionic carboxyl groups (–COOH, –COOCH_3_), arising mainly from carboxylic or carbonyl C=O linkages in acetyl ester groups. Bands in the range of 1652 cm^−1^ (No. 4), attributed to C=O or C=C stretching, and 1516 cm^−1^ (No. 5) were assigned to N–H deformation or C=N stretching; the band at 1247 cm^−1^ (No. 6) was attributed to C–N stretching; and that at 1055 cm^−1^ (No. 7) was assigned to the stretching vibration of C–OH of alcoholic groups and carboxylic groups.

Without DSE inoculation, compared with the control groups, the relative absorbance at bands No. 2, No. 3, No. 6, and No. 7 for the 5 mg·kg^−1^ Cd treatment decreased while it increased in DSE-inoculated plants contaminated with 5 mg·kg^−1^ Cd, suggesting that these related functional groups involved in the function of DSE affected adsorption in maize RCW. Without DSE inoculation, the FTIR spectra show that the peaks at bands No. 1, No. 4, and No. 5 shifted under three levels of Cd contamination when compared with control groups. Moreover, under 5 mg·kg^−1^ Cd conditions, the peaks at 3418, 1635, and 1569 cm^−1^ shifted to 3425, 1632, and 1559 cm^−1^, respectively, due to DSE inoculation.

### 3.6. Correlation Analysis

**Figure 6:**
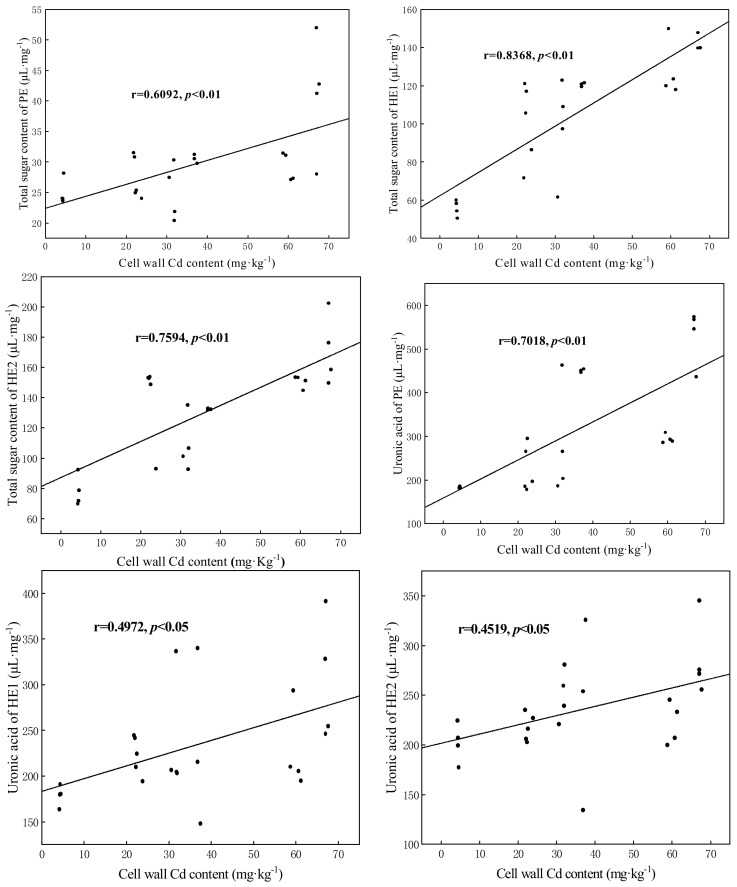
Correlation analysis between maize RCW Cd content and cell wall components. PE: pectin; HE1: hemicellulose–1; HE2: hemicellulose–2.

The correlation analysis shows that Cd content in the RCW of maize was highly significantly (*p* < 0.01) positively correlated with the total sugar content of PE, HE1, HE2, and PE uronic acid. Significantly (*p* < 0.05) positive correlations were observed between the cell wall Cd content and HE1 uronic acid and HE2 uronic acid (Figure 6).

## 4. Discussion

### 4.1. The Effects of DSE Inoculation on Growth and Cd Distribution of Maize

Recent studies show that DSEs are one of the most common root-associated fungi in metal-polluted soils and have low sensitivity to heavy metals [50]. In heavy metal contaminated soil, *E. pisciphila* widely colonizes plant roots, and due to the synergy of extracellular and intracellular mechanisms, the effects of DSE inoculation on plant growth are commonly positive under heavy metal stress [42,66,67], For instance, root-associated *E. pisciphila* promoted the growth of maize and improved the Cd tolerance of its plant partner, and their association is mutualistic under heavy metal stress [50,68]. Other DSE strains were also observed to promote host plant growth [69]. Therefore, it is possible that the colonization of *E. pisciphila* increased as a mutual response to increasing Cd toxicity, which enhances the fitness of both plants and fungi. This may represent an efficient strategy for surviving in a stressful environment. Despite this, there is some controversy regarding the harmfulness and neutral nature of DSE for plants [70,71]. Depending on the host plant’s physiology and genetic differences between cultivars, a single fungal isolate from a specific geographic area can act as a pathogen, mutualist, or commensalist [49,50,72,73,74]. The complex nature of the DSE–soil–plant system means that more data may be required from repeated experiments with different DSE–plant combinations to confirm DSE–plant interactions. Our experiment demonstrates that DSE colonization increased maize seedling biomass regardless of Cd addition. High concentrations of Cd exerted toxicity on maize, which severely reduced the biomass and plant height of maize, but DSE inoculation mitigated those negative consequences (Figure 1). This suggests that the increased Cd tolerance is involved in a changed mechanism of DSE–plant interactions and that host plants can benefit from DSE colonization and resist Cd uptake inside cells. Similarly, under Pb stress, inoculation with *Gaeumannomyces cylindrosporus* significantly increased maize height and the biomass of maize seedlings by improving the efficiency of photosynthesis and altering the translocation and accumulation of Pb in the plants. Pb mainly accumulates in the root system of maize and the phytotoxicity of Pb to the shoot is alleviated [75]. One of the reasons for that DSE colonization increases plant growth under metal addition is that DSE enhances metal tolerance and improves plant growth by altering metal repartitioning into the root and shoot [19,48,52,54]. In our results, we found that more Cd accumulated in maize with increasing concentration of Cd, and the Cd content in roots was higher than that in the shoots. Additionally, with DSE inoculation, the Cd sequestration in the roots was promoted while the translocation of Cd from the roots to the shoots was restricted (Figure 2). Li [50] found the same results. Similar to the results of this study, Single inoculation or coinoculation with AMF and DSE significantly decreased the Cd content in shoots and Cd transfer coefficient of maize [19]. However, He et al. [51] found that DSE colonization in roots significantly increased Cd accumulation in the shoots in 5 and 10 mg·kg^−1^ Cd treatments. Nevertheless, colonization with DSE significantly decreased Cd concentrations in maize (both the shoots and roots) in 50 and 100 mg/kg Cd treatments [52]. Additionally, during symbiosis, F. oryzae conferred and improved Cd tolerance to rice, decreasing Cd accumulation in roots and translocation to shoots [55]. We conclude that the impact of DSEs on heavy metal accumulation in host plants depends on the type and level of heavy metals in the substrate. The Cd distribution in different plant tissues influences its toxicity. Advantaged distribution is a crucial strategy in response to Cd stress in plants. Toxic ions can be isolated in specific areas where heavy metal ions are less active. We argue that the changes in the active status of Cd were of crucial significance for the enhanced tolerance of the DSE plants. The cell wall is the first barrier to stand against heavy metal and plays a pivotal role in Cd tolerance and accumulation [76]. The heavy metal in the cell wall is usually fixed in specific binding sites and the toxic effects are lower than the free ionic state situated in the cytosol and organelle [77]. Our results demonstrate that DSE inoculation significantly increased cell wall integrated-Cd with the elevated Cd stress, thus strongly limiting Cd accumulation in membranes.

In summary, colonization with DSE alters the transfer of heavy metals from the roots to the shoots of the host plant and many free ionic Cd molecules were fixed in the RCW which may serve as an effective tolerance strategy for maize to resist damage from Cd. However, the impact of DSE–plant interactions on plant growth and heavy metal content vary among strain type and change with Cd stress levels. Due to the complexity of the DSE–soil–plant system, more data from other repeated experiments within different DSE–plant combinations are needed to confirm DSE–plant associations.

### 4.2. The Effects of DSE Inoculation on RCW Polysaccharide Components of Maize

Researchers have confirmed the vital role of modulation of cell wall polysaccharide components in cellular responses to metal stress in plants [78,79]. Recent studies on two cultivars of *Brassica chinensis L.* showed that up to 79.4% and 32.1% of cell wall-bound Cd was found in PE and HE1, respectively [80]. Cell wall thickening is an apparent phenomenon in the modification of roots under toxic metal stress [81]. Such a modification may cause cell walls to be less permeable to Cd, limiting its entry into the cell. Earlier studies revealed that, under heavy metal stress, the total sugar and uronic acid content in the cell wall increase significantly [20,24,82,83]. Therefore, these findings suggest that carbohydrates exert a vital role in the metal binding of the cell wall. To determine whether DSE inoculation enhances the Cd retention in the cell walls of maize roots by modulating the carbohydrates of each cell wall component, we measured the total sugars and uronic acid in PE and HE in the roots. According to correlation analysis, we found that the greater Cd retention capacity of the RCW was closely related to the cell wall polysaccharide components. In our study, exogenous Cd altered the structures of cell wall components in maize roots. Under high Cd stress, the total sugar content of components in the RCW increased significantly which is consistent with the studies mentioned above. Without Cd treatments, DSE inoculation also significantly increased the total sugar content of cell wall components. Moreover, under Cd exposure, the DSE-inoculated plants resulted in a significant increase in the total sugarsHE1 and HE2 while a significant increase in PE total sugar was observed at 20 mg·kg^−1^ Cd stress. We argue that DSE colonization may bioaugment Cd sequestration in RCWs through increased carbohydrates. Thus, DSE colonization improves the Cd holding capacity of RCW by increasing the total sugar content of PE and HE, especially under high Cd stress.

However, the changes in the uronic acid content of cell wall components were different. Both DSE and Cd stress had a significant effect (*p* < 0.05) on the uronic acid content of cell wall components. Without DSE colonization, the difference in the Cd accumulation ability of RCW of maize is mainly reflected in the uronic acid of PE and HE1. The results are consistent with those of Wang et al. [80], who found that PE and HE1 are the principal sinks for Cd in RCWs. However, other findings show that only HE serves as the dominant binding site [84]. This contrary outcome may be due to the use of different plant species, experimental conditions, and different Cd concentrations. Under Cd stress, inoculation with DSE significantly improved the uronic acid content in cell wall components, especially in PE, suggesting that the PE fraction may be more sensitive to DSE inoculation under Cd stress. The increased total sugar and uronic acid may be applied to Cd fixation. Similar results were reported for *Medicago truncatula* under lead stress inoculated with arbuscular mycorrhizal fungi [25]. However, Cd stress or DSE inoculation did not significantly enhance the uronic content in HE2, indicating that the HE2 of DSE-inoculated roots and non-DSE roots has the same capacity to fix Cd through uronic acid.

To determine why PE and HE play such important roles in our research, further experiments and discussions are needed. Previous studies found that the cumulative carbohydrates induce an increase in low methyl-esterified PE to increase Cd immobilization [85,86]. Thus, we associate the capability of DSE inoculation to induce the strong fixation of Cd in maize RCW with adjustments in polysaccharide structure, mainly in the PE and HE fractions.

### 4.3. Effects of DSE Inoculation on Functional Group Changes

As noted above, the cell wall is the major binding site for Cd in many types of plants. The plant cell wall is composed of polysaccharides and proteins as well as several functional groups which are viewed as the main heavy metal ion binding sites [87]. Cell wall components are rich in hydroxyl, carboxyl, sulfhydryl, and other functional groups, which play important roles in ion exchange mechanisms [88,89,90]. For example, the fixation of divalent heavy metals was achieved by binding with the carboxyl groups of uronic acid in the cell wall [85]. Two free carboxyl groups can be combined (using Ca^2+^) and form a chelating structure, but divalent and trivalent ions (such as Cd^2+^ and Al^3+^) can replace Ca^2+^ in the structure [86]. DSE strains of *Exophiala pisciphila* upregulated the expression of genes involved in cell wall biosynthesis and changes in the content of functional groups in PE and HE1 in response to Cd stress [76]. Functional groups of PE can combine with Cd^2+^ and Al^3+^ in *Sedum alfredii* and *Medicago sativa* to increase the Cd tolerance of the host plants [91,92].

The FTIR results further show that DSE colonization changes the peaks of carboxyl, hydroxyl, and acidic groups in the cell wall, indicating that these groups are more pronounced functional groups involved in Cd sequestration in maize RCW when inoculated with a DSE. After the increases in these groups in the cell wall, the Cd immobilized in the cell wall increases. This changes the distribution of metals in maize roots, prevents the toxic ions from entering the cytoplasm, reduces the phytotoxicity of metals in different root organelles, reduces the transfer of metals to shoots, and promotes Cd retention in the root system.

## 5. Conclusions

Under Cd stress, inoculation with a DSE alleviated the stress of heavy metals on maize growth and modulated RCW polysaccharide components, which enhanced Cd retention in the roots while decreasing Cd accumulation in the shoots. The correlation analysis results show that the greater Cd retention capacity of the RCW is closely related to the increased content of cell wall polysaccharide components which provide binding and adsorption sites for heavy metals. DSE inoculation significantly increased the total sugar content of PE and HE in RCW components while an increase in uronic acid was only observed in PE, indicating that HE2 of DSE inoculation roots and non-DSE roots has the same capacity to fix Cd through uronic acid. In addition, in the DSE-inoculated roots, functional groups, such as carboxyl, hydroxyl, and acidic groups, complexed with Cd^2+^, fixed Cd in the RCW and effectively reduced the toxicity of Cd to plants to promote the continuous growth and development of cells. In conclusion, the results of this study elucidate the roles of DSEs in promoting Cd retention in host plant roots.
